# Community resilience to health emergencies: a scoping review

**DOI:** 10.1136/bmjgh-2024-016963

**Published:** 2025-04-12

**Authors:** Gisela van Kessel, Steve Milanese, Janine Dizon, Daniel H de Vries, Hayley MacGregor, Sharon Abramowitz, Luisa Enria, Doris Burtscher, Eng-Kiong Yeoh, Beena E Thomas, Rim Kwang, Joao Rangel de Almeida, Nina Gobat

**Affiliations:** 1 UniSA Online, University of South Australia, Adelaide, South Australia, Australia; 2 School of Health Science, Swinburne University of Technology, Melbourne, Victoria, Australia; 3 University of South Australia Allied Health & Human Performance Academic Unit, Adelaide, South Australia, Australia; 4 ARIIA, Flinders University, Adelaide, South Australia, Australia; 5 University of Amsterdam, Amsterdam, Netherlands; 6 Health and Development, Institute of Development Studies British Library for Development Studies, Brighton, UK; 7 Rutgers The State University of New Jersey, New Brunswick, New Jersey, USA; 8 Global Health and Development, London School of Hygiene and Tropical Medicine, London, UK; 9 Medecins Sans Frontieres, Geneva, Switzerland; 10 The Jockey Club School of Public Health and Primary Care, The Chinese University of Hong Kong, Sha Tin, Hong Kong; 11 Department of Social and Behavioral Research, National Institute for Research in Tuberculosis, Chennai, Tamil Nadu, India; 12 Country Readiness Strengthening Department, World Health Organization, Geneva, Switzerland; 13 World Health Organization, Geneva, Switzerland

**Keywords:** Global Health

## Abstract

**Background:**

There is recognition of the importance of community resilience in mitigating long-term effects of health emergencies on communities. To guide policy and practice, conceptual clarity is needed on what community resilience involves and how it can be operationalised for community protection in ways that empower and strengthen local agency.

**Objectives:**

To identify the core components of community resilience to health emergencies using a scoping review methodology.

**Search methods:**

PubMed, EMCARE, Scopus, Web of Science, PTSDpubs, APO and ProQuest Dissertations were systematically searched to identify review studies published from 2014.

**Selection criteria:**

Studies were included if they reported a review of original research papers investigating community resilience in the context of a health emergency.

**Data collection and analysis:**

Data were extracted from included studies using a specially developed data extraction form. Qualitative data were subjected to a meta-synthesis consisting of three levels of analysis.

**Main results:**

38 evidence reviews were included. Analysis identified recurring characteristics of community resilience. Six studies reported 10 abilities required for community resilience including: adapt, transform, absorb, anticipate, prepare, prevent, self-organise, include, connect and cope. 25 studies reported 11 types of resources: social, economic, environmental, governance, physical infrastructure, institutional, communication, human capital, health, emergency management and socioeconomic.

**Conclusions:**

21 components have been identified that can be used as a basis for operationalising and measuring community resilience. In contexts of disaster management, community resilience is a fairly mature concept that reflects a community’s inherent capacity/abilities to withstand and recover from shocks. There is a need to incorporate a ‘resource’ perspective that speaks to a wider enabling environment. There is scope to investigate whether the same set of components identified here has relevance in public health emergencies emanating from disease or human acts of aggression and to articulate resilience logics to critical endpoints for health emergency management.

**WHAT IS ALREADY KNOWN ON THIS TOPIC**

**WHAT THIS STUDY ADDS**

- The process of community resilience includes the ability of the community to adapt, transform, absorb, anticipate, prepare, prevent, self-organise, include, connect and cope in response to a hazard.
- However, these abilities rely on the existence of adequate resources, both in type and amount.
- Adequate resources for community resilience should encompass sufficient social capital, physical infrastructure, economic, environmental, governance, institutional, communication, health, human capital, emergency management and socioeconomic resources.
- Effective operationalisation of community resilience in response to a health emergency relies on adequate resources available to a community with the ability to access and use them effectively.

**HOW THIS STUDY MIGHT AFFECT RESEARCH, PRACTICE OR POLICY**

- Further research is needed to explore the factors that enable the development of the ability of a community for resilience, including what hinders its development and how it might be constrained during an emergency.
- Further work could develop a consensus on a theoretical model of community resilience that encompasses the complex, multiple interlinkages between different abilities and resources across different health emergency contexts.

## Introduction

The past decade has seen multiple national and international health emergencies, from the international spread of wild poliovirus in 2014, the Ebola epidemic in West Africa from 2014 to 2016 and the Democratic Republic of the Congo from 2018, the Zika virus epidemic of 2016, the H1N1 pandemic in 2019 and the ongoing COVID-19 pandemic. In addition, postdisaster disease outbreaks have been linked to conflicts and hydrological events commonly caused by bacterial and waterborne agents.^1^ Definitions of health emergencies vary in the literature but are considered here as any occurrence or imminent threat to a community of widespread or severe damage to health, injury or loss of life resulting from a natural phenomenon or human act.^2^ Public Health Emergency of International Concern (PHEIC) is defined as constituting ‘an extraordinary event’ that is ‘a public health risk to other states through the international spread of the disease’.^3^ Such events require a coordinated immediate international response for a situation recognised as serious or unexpected’.^4^ The concept of a PHEIC is not limited to epidemic-prone diseases but extends to biological, chemical and nuclear hazards, including the chemical or nuclear contamination of the environment, and contaminated food and pharmaceuticals. Far more common than these extraordinary PHEIC events are the hundreds of small to medium events that affect communities worldwide and are dealt with at the local level.

Public health emergencies are increasing in size, scale and scope.^5^ In 2023, WHO responded to 65 graded, acute and protracted health emergencies.^6^ Drivers of these events are complex and include geopolitical and national conflict, change in climate, food insecurity, weakened health systems, and widening health, social and economic inequalities. The widespread and obvious devastating long-term effects of these events on local communities have seen an increasing interest in the concept of strengthening community resilience in (anticipation of) response to health emergencies. When faced with a public health emergency, local communities are the first to be directly impacted and often represent the first line of defence in terms of disaster response.^7^ There is, therefore, an ethical and practical imperative to engage with those at risk or affected for effective and community-centred health emergency management.^5^ Health and resilience are considered interdependent, with health being integral to a community’s resilience^8^ and a community’s resilience underpinning its response to a health emergency. This concept of community resilience has been recognised and consequently embedded in multiple frameworks for disaster risk management, including the Sendai Framework for Disaster Risk Reduction (2015–2030) and the Health Emergency and Disaster Risk Management Framework (2019). For the purposes of this review and in the context of a public health emergency, we define communities as groups of people linked by common features such as identity, geography, age, gender, ethnicity, occupation, commitment, interest or concern that are affected by the emergency event.^9 10^ Communities are heterogeneous and differ in certain aspects, including culture and beliefs and other characteristics. They have unique social structures, forms of authority and representation, and power dynamics both internally and with other communities.^11^ Communities can be defined at multiple levels, including local, subnational, national, regional and international levels.

The focus on community resilience to health emergencies reflects the increasing awareness that affected communities have an intrinsic capacity to respond to health crises. This is distinct from an individual’s resilience or an institutionalised response to health emergencies. For example, affected communities to varying extents have formal and informal structures for rapid communications, social networks for resource sharing, highly localised knowledge about individual and household vulnerabilities, deep local knowledge of local environmental, health, economic and political systems and their reactivity, and ability to negotiate with local leaders and stakeholders. The ways in which public health actors view and understand these resilience factors will influence the kinds of partnerships, programmes and interventions that they design and implement in the well-recognised cycle of prevention, preparation, readiness, response and recovery to health emergencies.

The United Nations International Strategy for Disaster Reduction defines resilience as ‘the ability of a system, community or society exposed to hazards to resist, absorb, accommodate to and recover from the effects of a hazard in a timely and efficient manner, including through the preservation and restoration of its essential basic structures and functions’.^12^ This definition describes community resilience as the relationship between a community’s response to a health emergency hazard as one of resistance, absorption and accommodation and the desired outcome in relation to the community’s structures and functions. In policy and academic publications, a range of definitions of community resilience exists, reflecting the complex nature of this concept. This conceptual ambiguity presents challenges to building a reliable evidence base and operationalising it in meaningful ways.^13^ Further, policy discourse on community resilience has also not gone unchallenged. For example, critiques have argued that emphasis on community resilience shifts responsibility for community protection from the state to groups of individuals, bypassing the need to consider local realities and wider systemic drivers of vulnerability and inequities and inequalities.^14^ Others have contested the transferability of resilience, which is an ecological concept that refers to ways in which natural environments absorb disturbances, recover from stressors and maintain their essential functions, to human communities.^14^ The focus on community resilience also often fails to recognise that resilience requires human, financial and logistical resources, which erode over time in pandemic conditions.

Despite these shortcomings, community resilience is a central frame used in many policies and frameworks that aim to strengthen global architecture to withstand and recover from emergency events.^5^ For this concept to be of value to guide policy and practice, key questions need addressing as to what exactly community resilience involves and how it can be operationalised for community protection and recovery during public health emergency events in ways that empower communities and strengthen local agency.^5^ Resilience-oriented policy development and implementation involves nuanced processes, influenced by political contexts and with unintended consequences that need critical reflection and awareness. To assist in developing a greater understanding, this paper aims to identify the core components of community resilience to health emergencies, based on a review of the literature.

## Materials and methods

Due to the complex nature of community resilience in health emergencies, we conducted a scoping review to synthesise the evidence related to this concept. A number of evidence reviews have explored community resilience in cases of health emergencies; however, most have focused on specific health emergencies (ie, the COVID-19 pandemic),^15^ specific outcome measures^16^ or specific communities.^17^ Our explicit objective was to synthesise the evidence related to the core components of community resilience to all health emergencies. Scoping reviews are recommended when the aim of the review is to map the key concepts underpinning a topic rather than focus on a relatively precise question.^18^ To ensure the relevance of the review, an expert reference group was convened that included members with experience in working with communities during health emergencies. The use of expert reference groups in the development of evidence reviews has been promoted to improve their relevance.^19^

The protocol underpinning this review was registered with Open Science Framework (DOI 10.17605/OSF.IO/CWNU8)

### Study selection criteria

We initially constructed the search to explore all original research reports, including both primary and secondary research. When we reviewed the initial search results, we found a high number of articles identified on the topic of community resilience and multiple evidence reviews. We decided to restrict the scoping review to evidence from secondary evidence sources, such as systematic review, scoping review and narrative reviews. This focused the analysis on exploring and summarising the concept of community resilience to health emergencies, and not a review of evidence of effectiveness of interventions. Summarising the information from multiple overview articles would allow a clearer and more extensive overarching view on what constituted community resilience.

We included review studies that reported on community resilience in health emergencies and were published in 2014. This year limit was implemented based on the commencement of the Sendai Framework for Disaster Risk Reduction 2015–2030. This decision was supported by the findings of initial scoping searches which identified a significant increase in publications from 2014.

We included reviews based on the following criteria, which were guided by the expert reference group.

1. Reviews that reported on any hazard, in any country and setting, which may contribute to a health emergency, in the context of communities described as people living within a specific geographical area, involved in ongoing social interaction and with psychological ties to each other and to the place where they live.
2. Reviews that reported on any related aspect of community resilience in response to public health emergencies such as community resilience programmes.

We excluded studies if they reported on individual human resilience, were not published in English language or were not available to the reviewers in full text.

### Data sources and searches

**Figure 1:**
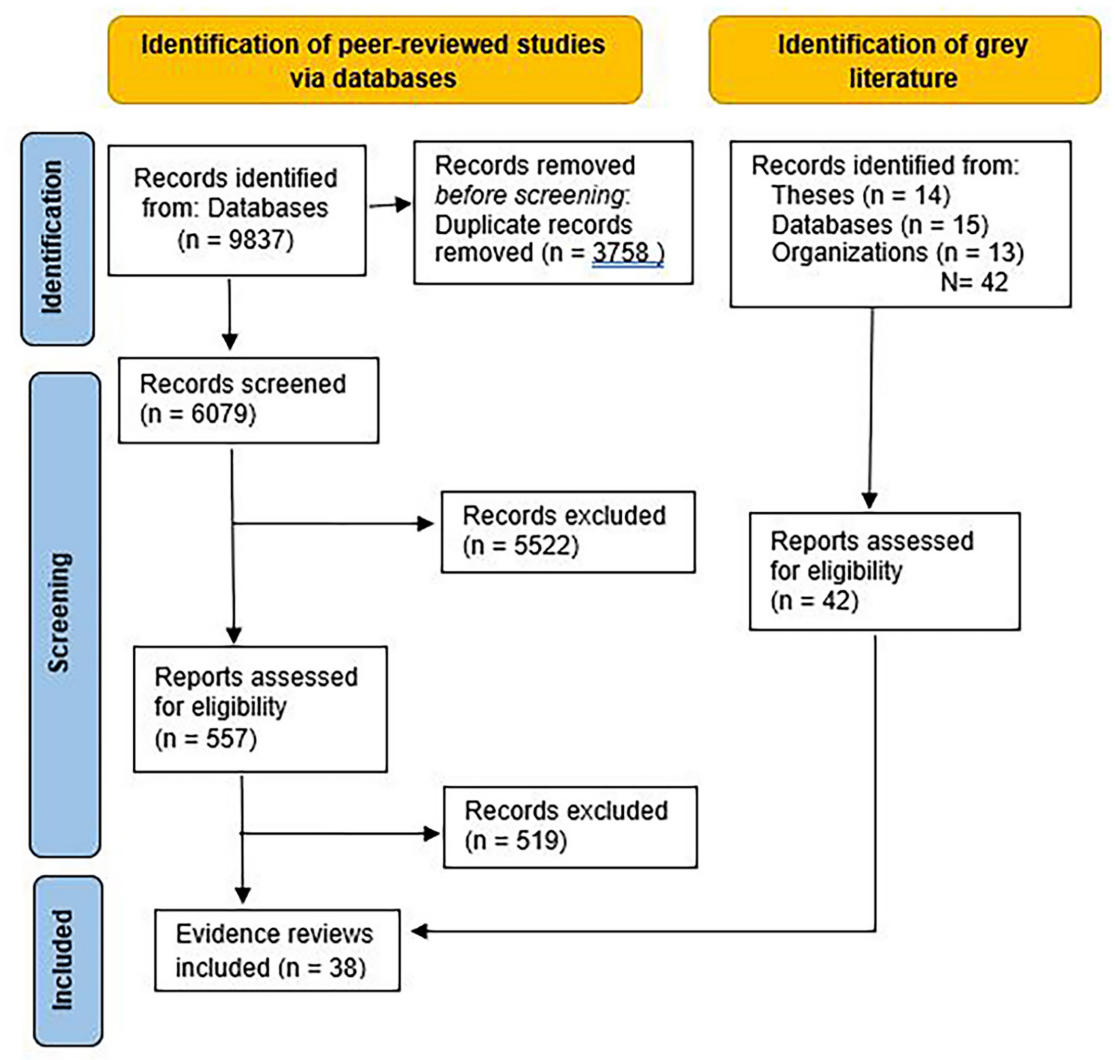
The process of screening that began with 9837 records identified from the databases and resulted in 38 studies included in this scoping review.

The search is presented in figure 1.

We searched for original research and reviews in November 2023 in the following electronic databases: PubMed, EMCARE, Scopus, Web of Science and PTSDpubs. Grey literature searches were undertaken using APO (Analysis and Policy Observatory) and ProQuest Dissertations and Theses Global databases. Searches were also undertaken of government agencies, peak bodies, relevant research institutes and websites of relevant agencies (WHO, UNICEF, IFRC, RAND corporation, Cordaid Institute for Sustainable Communities).

The search strategy for the MEDLINE database search is available in online supplemental appendix 1. Search terms were developed in conjunction with an independent academic librarian. The MeSH keyword search terms and Boolean operators were modified to accommodate each search database.

### Study selection

All database searches were transferred to EndNote and duplicates removed. Endnote files were then transferred into Covidence software for title and abstract screening. One researcher conducted the evidence search, and two researchers independently screened title and abstract and then assessed full text.

### Data collection and synthesis

A standard data extraction form was developed to extract data from all included studies. Prior to data extraction, a random subgroup of 10 articles was reviewed by the review team and responses examined for consistency and comprehension. The data extraction process was undertaken independently by two reviewers. Reviewers consulted other reviewers in the team if there was anything that needed verification in the data extracted.

Extracted data included the following:

- Author(s).
- Year of publication.
- Evidence type (systematic review, scoping review, narrative review).
- Aims/purpose.
- Community description.
- Type of public health emergencies.
- Descriptions from included publications on the core components/elements of community resilience programmes.

The SIGN (Scottish Intercollegiate Guidelines Network) checklist, which was specific to the study design of the included studies, was used to assess the methodological quality of the studies. The relative methodological quality of the reviews was used to gauge the relative risk of bias associated with each review. A risk of bias score was developed with reviews scoring 7 or greater on the SIGN checklist being rated as low risk of bias (L), between 6 and 4 moderate risk of bias (M) and reviews scoring 3 or less being rated as a high risk of bias (H). This risk of bias score provided a guide on the relative methodological strength of each review.

A qualitative thematic analysis was conducted to develop categories by sorting findings related to the characteristics of components or elements of community resilience programmes. Data from the included studies in the data extraction form were coded. Codes were sorted based on characteristics of community resilience to identify components.

## Results

### Characteristics of the included studies

The characteristics of the 38 included studies are summarised in table 1. Six studies reported systematic reviews, 19 described scoping reviews and 11 reported narrative reviews. Of these, 1 was of low risk of bias, 19 were of moderate risk of bias and 16 were at high risk of bias. As expected, narrative reviews were at the highest risk of bias due to a lack of a reported literature search methodology or consideration of the strength of the included evidence.

**Table 1:** Reviews exploring the core components of community resilience in health emergencies

Study	Review type	Studies	Risk of bias
van Kessel *et al*^20^	Systematic review	8	Moderate
Xu *et al*^21^	Systematic review	324	High
Amirzadeh *et al*^22^	Systematic review	97	Moderate
Carvalhaes *et al*^23^	Narrative review	Not reported	High
Feldmeyer *et al*^24^	Narrative review	Not reported	High
Koren *et al*^25^	Narrative review	Not reported	High
Ningrum and Subroto,^26^	Scoping review	47	Moderate
Rus *et al*^27^	Scoping review	Not reported	Moderate
Zhang and Wang^28^	Narrative review	Not reported	High
Almutairi *et al*^29^	Scoping review	64	High
Heagele^30^	Scoping review	Not reported	High
Maulana and Wardah^31^	Narrative review	Not reported	High
Olimid *et al*^32^	Narrative review	Not reported	High
Saja *et al*^33^	Scoping review	31	Moderate
Tariq *et al*^34^	Scoping review	Not reported	Moderate
Zamboni^35^	Scoping review	19	Moderate
Cui *et al*^36^	Systematic review	71	Moderate
McClelland *et al*^37^	Narrative review	Not reported	High
Rela *et al*^38^	Narrative review	Not reported	High
Suleimany *et al*^39^	Systematic review	115	Moderate
Zhang *et al*^40^	Narrative review	Not reported	High
Manyena *et al*^41^	Narrative review	Not reported	Moderate
Patel *et al*^42^	Scoping review	80	Moderate
Sharifi and Yamagata^43^	Scoping review	Not reported	High
Mochizuki *et al*^44^	Systematic review	Not reported	Moderate
Ribeiro and Gonçalves^45^	Scoping review	83	Moderate
Saja *et al*^46^	Scoping review	31	Moderate
Nguyen and Akerkar^47^	Scoping review	77	Moderate
Assarkhaniki *et al*^48^	Scoping review	Not reported	High
Cai *et al*^49^	Scoping review	174	High
Kamara *et al*^50^	Systematic review	19	Low
Cutter^51^	Narrative review	Not reported	High
Meng *et al*^52^	Scoping review	Not reported	Moderate
Summers *et al*^53^	Scoping review	Not reported	Moderate
Koliou *et al*^57^	Narrative review	Not reported	High
Jewett *et al*^60^	Scoping review	Not reported	Moderate
Pfefferbaum *et al*^61^	Narrative review	Not reported	High

Included studies based their conclusions on reviews ranging from 8 papers^20^ to 324.^21^ Eight studies reported emergencies in urban communities^21^,^28^ and one on coastal events.^29^ 12 reviews investigated public health emergencies in the context of natural disasters.^2021 23^,^25 29^ 10 papers investigated community resilience in the context of a pandemic.^2226 28 32 35^,^40^

**Figure 2:**
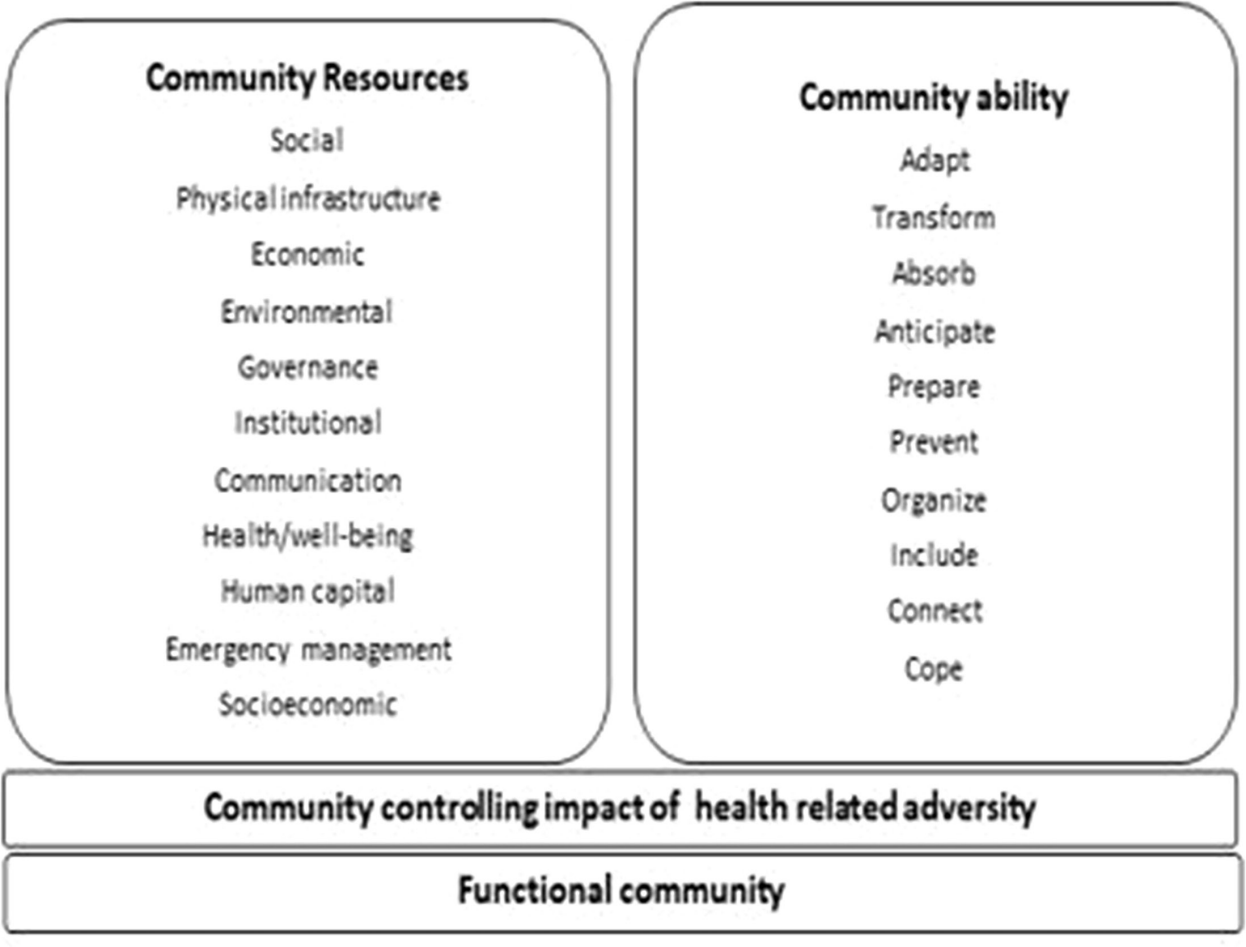
Community resilience to health-related adversity.

The analysis identified two key elements of community resilience to health emergencies (see figure 2). First, abilities describe the capability or capacity of a community for resilience (see online supplemental appendix 2, online supplemental material). Second, the resources required for community resilience (see online supplemental appendix 3, online supplemental material).

### Abilities as components for community resilience

10 community abilities observed to be required for resilience to a health emergency were extracted from 13 of the identified studies (online supplemental appendix 2, online supplemental material). The studies suggest that the abilities to adapt, connect, transform, self-organise, anticipate, prepare, prevent, absorb, include and cope are observed in communities that are more likely to demonstrate resilience.

The four most cited abilities include adaptation, connection, transformation and self-organisation, with adapting as the most commonly reported ability.^21^ Adapting occurs as communities learn,^41^,^43^ plan,^43^ innovate^43 44^ and adjust to risk factors.^44^ Adapting enables improvisation and replacement of affected systems.^30 41 45^ Connected communities coordinate and share goals, resulting in an exchange of information and stronger decision-making.^22 23 30 37 42 45 46^ Transforming actions help communities to reconfigure or modify ecological, economic and social structures and risks.^23 37 41 44 46^ Self-organising communities require minimal outside assistance, relying on good social capital and social learning resources.^22 23 30 37 41 45^

Three abilities were described as relevant to the prevention phase of emergency management, that is, the ability to anticipate, prepare and prevent. Anticipating occurs through activities that gather information such as scanning, forecasting and predicting and understanding risk.^41^ Communities who undertake preparation gather and use information to determine their baseline infrastructure resources and are aware of vulnerable groups within the community.^30 43^ They also prepare by conducting risk assessments^42^ and establishing partnerships that can work to avoid or resist the negative effects of a health emergency event.^30 43^ While communities who have the ability to anticipate and prepare foresee what is required to manage the impacts of emergencies, some communities have the ability to prevent and mitigate negative outcomes by taking actions to avoid risks both before and during a health emergency event.^41^

The remaining three abilities help a community to respond and include absorption, inclusion and coping. Absorbing occurs as communities persist and persevere in their immediate response to isolate the disruption.^41 43^ Inclusive communities consult and involve all members of the community in decision-making processes to ensure resources are allocated equitably to the most vulnerable.^23 30 45 47^ Finally, coping is described in three reviews as the ability of a community to maintain equilibrium or bounce back to a predisaster level of functioning, but there is a lack of distinction between individual and community coping.^34 41 44 46^

### Resources as components for community resilience

11 resources required to operationalise community resilience were identified in 34 studies included in this scoping review. Resources were categorised as sociocultural, physical infrastructure, economic, governance, environmental, institutional, communication, health, human capital, emergency management and socioeconomic (online supplemental appendix 3, online supplemental material).

Social resources were identified as a main component of community resilience, being reported in 30 out of 35 papers. Social resources were described as social networks, social capital, social cohesion, social support and cultural capital.

Physical resources included any technology and infrastructure in the built environment and involve a network of essential structures, including private and public buildings that provide services (ie, hospitals, banks, schools),^3948^,^50^ services provided to buildings—such as communication, power, water, sanitation,^25 29 42 48 51 52^ digital infrastructure,^26^ transport,^29 39 48 49^ shelter,^39 48 49^ as well as structures designed to control hazards such as dams^42^ or indoor ventilation.^22^

Economic resources are found within the financial resources of governments,^32 48^ businesses^34^ and individual or household wealth.^34 41^ Both the amount and diversity of economic resources distribution contribute to a community’s resilience.^34 38 39 41^

Governance and institutional resources are intertwined. Governance is a resource enacted with multiagency collaboration,^34 37 53^ leadership^28 34 42 52^ and partnerships,^34 42^ through laws, regulations, disaster and emergency plans, policies and training and the management of natural resources.^34^ Institutional resources include organised governmental services, resource management, warning and evacuation procedures, emergency response and disaster recovery.^35 39 40 45 48 49^

Environmental resources are natural or ecological resources such as the quality and diversity of food; the quality of land and soil; air and water; biodiversity; environmental policies regarding energy; environmental services such as recycling; waste management; and recreational open spaces.^22 25 34 38 48^

Resources related to the members of the community include communication, health and human capital. Effective communication and use of information resources relied on digital infrastructure, diverse modes of communication and types of content that align with community perceptions delivered in real-time communication during a crisis,^26 38 42^ as well as supporting equitable access to people who cannot access technological forms of communication.^20^ Health resources include the status of the population, the provision of health services and health infrastructure.^42^ Healthy communities require services with sufficient trained personnel,^43 46^ infrastructure and good governance.^34^ Communities rely on a population of individuals that has sufficient self-efficacy and empowerment,^42^ can provide skills, labour,^25 50^ is literate in health,^34 42 48 50 52^ draws on local knowledge^42^ and has economic capacity.^39 48 50 53^

Less frequently cited are emergency management experience and socioeconomic resources. Prior emergency management experience provides learnings from the past that can be a key resource contributing to increased preparedness (and thus enhanced capability).^23 24 30^ Socioeconomic resources include the role of population characteristics, demographics, health, education, employment, income, community and household capacity for income generation.^30 48^

## Discussion

This scoping review identified 38 studies that described 21 components within two key elements of community resilience to health emergencies. Due to the nature of the current evidence, the components relate more strongly to community resilience to natural disasters. There is comparatively less evidence regarding public health emergencies emanating from disease or human acts of conflict. The review findings indicate that community resilience is operationalised by the ability of a community to access and use its resources in order to experience resilience in response to experiencing a hazard. Resilient communities have a capacity for collective action that uses the community’s resources to solve collective problems. This includes the capacity to adapt, transform, absorb, anticipate, prepare, prevent, self-organise, include, connect and cope. However, these abilities rely on the existence of adequate resources, both in type and amount. Conversely, a community that has adequate social capital, physical infrastructure, economic, environmental, governance, institutional, communication, health, human capital, emergency management and socioeconomic resources still needs to have the capability to access and use them. Further research is needed to explore the factors that enable the development of capacity, what hinders its development and how it might be constrained during an emergency. Community resilience is not something that communities simply have or don’t have, but rather, reflects a set of conditions that can be developed, and that doing so requires a much broader and deeper approach that considers acting on structural factors far beyond the emergency itself, and requires active commitment by governments/multilateral agencies to be fostered. Of note is that, beyond reference to emergency management experience, the role of previous events or history did not emerge much in this review, and the role of institutional and cultural memory as such is undervalued.^54^

How a component is defined was observed to vary across the literature included in this review. Components were described as properties, processes, domains, dimensions or elements and often linked to subcategories, referred to as subdomains or indicators. A component was included in this analysis if it was distinctly different from other elements and was validated by at least one other author. Codes that appeared only once were included within similar categories, for example, poverty was included in the socioeconomic category,^34^ quality of life was included in the health category,^34^ and technical was included in infrastructure.^27^ Elements of robustness, redundancy and rapidity were also described in the literature,^30 43 45^ but they were conceptualised as ways of measuring the dynamic use of resources.^55^ In the same way, the process of recovery appears as an outcome of community resilience.^43^

The identification of 21 components of community resilience in this review aligns with framings of community resilience as a complex systems-level concept. Community resilience arises through the interaction and interconnections between components. The amount of interconnectivity and the interactions that are critical will vary according to context, that is according to a broader set of geographic, sociocultural, historical, epidemiological, socioeconomic, political and other characteristics and circumstances of both the communities and the public health emergency. These relationships between components are fluid and dynamic: the interaction between communities’ resources and abilities will change over time and are influenced by communities’ past and current experiences of the adverse event itself.^50 56^ Further complexity arises because of the fluid and dynamic quality of the resources and abilities themselves. During a public health emergency, resources and abilities may fluctuate in their diversity, efficiency, robustness, redundancy or rapidity.^48 57 58^

This dynamic nature of community resilience has been increasingly recognised, with authors identifying the need for integrated approaches across multiple social, ecological, economic and technological domains of resilience.^59^ This dynamic complex nature of community resilience has seen the emergence of resilience models such as the resilience of social-ecological-technological systems.^59^ Despite the extent of interest in the field of resilience, there remains a lack of consensus on a theoretical model that encompasses the complex, multiple interlinkages between different abilities and resources across different dimensions of resilience in a community.

As with all published reviews, there are limitations to this review. The studies included in this scoping review were limited to English language studies only. While the reported health emergencies occurred in a wide range of countries, including non-English language countries, the focus on English language studies, for pragmatic reasons, is a limitation. Another limitation is the inclusion of a range of review types, most of which do not report on the quality of the included studies. Likewise, as this was a scoping review, a critical appraisal of the included studies was not undertaken. As the aim of a scoping review is to identify the core components of community resilience to health emergencies, and not to judge the effectiveness of an intervention and develop recommendations, the need for study quality appraisal is not paramount. The scope of this review did not explore a number of implications, and so there is a need for more research to examine the interactions and the interconnections of components and what the barriers and enablers to community resilience are. Furthermore, only reviews that identified the emergency as a public health emergency were included, which may affect the generalisability to other emergencies.

A strength of this scoping review is the broad umbrella perspective taken of the topic. This inclusion of all types of health emergencies allows for a big picture view of what community resilience may involve. The inclusion of an expert reference group to help guide the protocol development maximises the relevance of the review findings.

## Conclusions

This scoping review found core 21 components of community resilience to public health emergencies. The thematic analysis of the 38 reviews identified two key elements. Abilities are the observable capabilities or capacities of a community for resilience and include the ability to adapt, transform, absorb, anticipate, prepare, prevent, self-organise, include, connect and cope. The element of resources describes the measurable components required for community resilience and includes social, physical infrastructure, economic, environmental, governance, institutional, communication, health, human capital, emergency management and socioeconomic.

## Supplementary material

10.1136/bmjgh-2024-016963 online supplemental file 1

## Data Availability

Data are available on reasonable request.
