# Supplementary material for: Community resilience to health emergencies: a scoping review
Source: BMJ Glob Health. 2025 Apr 12;10(4):e016963. doi: 10.1136/bmjgh-2024-016963 (PMC11997830; doi:10.1136/bmjgh-2024-016963)
Supplement: online supplemental file 1 [file bmjgh-10-4-s001.pdf]

Supplementary Material

Appendix 1: Medline Search strategy

Search terms - Community Resilience	Search terms - Emergency	Limits
Resilien*, Psychological/ and (community or communities or town* or village* or suburb* or neighbourhood* or borough* or shire* or council or councils).ti,ab.<br>• Community Participation/<br>• Community response/<br>• Community Empowerment<br>• ((community or communities or urban or urban slum or cities or city or diaspora or town* or village* or suburb* or neighbourhood* or borough* or shire* or council or councils) adj5 (participat* or involv* or action* or resilien* or cohesion)).ti,ab,kf.	Disasters/<br>Disaster Planning/<br>Emergencies/ and (health* or plan* or prepared* or respons* or readiness).ti,ab.<br>Mass Casualty Incidents/<br>exp Natural Disasters/<br>Radioactive Hazard Release/<br>(disaster* or flood* or tsunami* or tidal wave* or drought* or fire* or wildfire* or bushfire* or bush fire* or typhoon* or hurricane* or tornado* or cyclon* or earthquake* or avalanche* or landslide* or volcano* or (nuclear adj3 (accident* or fallout or meltdown))).ti,ab,kf.Public Health/ and (disaster* or flood* or tsunami* or tidal wave* or drought* or fire* or wildfire* or bushfire* or bush fire* or typhoon* or hurricane* or tornado* or cyclon* or earthquake* or avalanche* or landslide* or volcano* or (nuclear adj3 (accident* or fallout or meltdown))).ti,ab.<br>Climate change and environmental disaster/<br>Disease Outbreaks/<br>Epidemics/<br>Pandemics/<br>COVID-19/<br>Hemorrhagic Fever, Ebola/<br>Coronavirus Infections/<br>Severe Acute Respiratory Syndrome/<br>Influenza, Human/<br>Influenza A Virus, H1N1 Subtype/<br>Middle East Respiratory Syndrome Coronavirus/<br>Zika Virus Infection/<br>Zika Virus/<br>Communicable Diseases, Emerging/<br>(outbreak* or infectious disease* or epidemic* or pandemic* or ebola* or covid* or coronavirus* or MERS or zika or influenza* or SARS or respiratory syndrome*).ti,ab,kf.<br>Health crisis<br>Health threats	Limit to year: 2014 - Current

Appendix 2. Abilities as Components for Community Resilience

	Adapt	Connect	Transform	Self organise	Include	Prepare	Anticipate	Prevent	Absorb	Cope
Amirzadeh et al, [22]	x	x		x						
Carvalhaes et al, [23]	x	x	x	x	x					
Heagele, [30]	x	x		x	x	x				
Manyena et al, [41]	x		x	x			x	x	x	x
McClelland et al, [37]	x	x	x			x				
Mochizuki et al, [44]	x		x							x
Nguyen & Akekar, [47]	x				x					
Patel et al, [42]	x	x				x				
Ribeiro & Goncalves, [45]	x	x		x	x					
Saja et al, [33]	x	x	x							x
Sharifi & Yamagata, [43]	x					x			x	
Tariq et al., [34]										x
Xu et al [21]	x					x				

Appendix 3: Resources as Components for Community Resilience

Review	Social/ Cultural	Physical infrastructure	Economic	Governance, policy and planning	Environmental	Institutional	Communication	Health/ wellbeing	Human capital	Emergency management	Socioeconomic
Almutairi et al, [29]	x	x	x	x	x	x					
Amirzadeh et al, [22]		**x**	x		x						
Assarkhaniki et al, [48]	x	x	x	x	x	x		x	x		x
Cai et al, [49]	x	x	x		x	x					
Carvalhaes et al, [23]	x	x	x		x	x	x			x	
Cui et al, [36]	x										
Cutter [51]	x	x	x		x	x	x			x	
Feldmeyer et al, [24]	x	x	x	x	x						
Heagele [30]								x		x	x
Jewett et al [55]	x										
Kamara et al, [50]	x			x					x		
Koren et al, [25]	x	x			x		x				
Koulio et al, [54]	x	x	x								
Maulana et al, [31]	x										
McClelland et al, [37]				x							
Meng et al, [52]	x	x	x	x					x		
Nguyen & Akekar [47]	x	x	x	x			x	x			
Ningrum et al, [26]	x	x	x	x			x				
Olimid et al, [32]	x	x		x							
Patel et al, [42]	x		x	x			x	x	x		
Pfefferbaum et al, [56]	x						x				
Rela et al, [38]	x	x	x	x	x				x		

Review	**Social/ Cultural**	**Physical infrastructure**	**Economic**	**Governance, policy and planning**	**Environ mental**	**Instituti onal**	**Communi cation**	**Health/ wellbeing**	**Human capital**	**Emergency management**	**Socioecon omic**
Ribeiro & Gonçalves [45]	x	x	x		x	x					
Rus et al, [27]	x	x	x	x	x	x		x	x		
Saja et al, [33]	x										
Saja et al, [46]	x						x	x			
Sharifi and Yamagata, [43]	x	x	x		x	x					
Suleimany et al, [39]	x	x	x			x		x			
Summers et al, [53]	x	x		x	x						
Tariq [34]	x	x	x	x	x			x	x		
van Kessel et al, [20]	x						x				
Xu et al, [21]											
Zamboni [35]	x	x	x			x					
Zhang et al,[40]	x					x		x			
Zhang et al, [28]	x		x			x	x		x	x	
